# Linking childhood trauma to the psychopathology of schizophrenia: the role of oxytocin

**DOI:** 10.1038/s41537-024-00433-9

**Published:** 2024-02-22

**Authors:** Yuan-Jung Chen, Mong-Liang Lu, Yi-Hang Chiu, Chenyi Chen, Vitor Hugo Jesus Santos, Kah Kheng Goh

**Affiliations:** 1 Department of Psychiatry, Wan Fang Hospital, Taipei Medical University, Taipei, Taiwan; 2 Psychiatric Research Center, Wan Fang Hospital, Taipei Medical University, Taipei, Taiwan; 3 Department of Psychiatry, School of Medicine, College of Medicine, Taipei Medical University, Taipei, Taiwan; 4 Graduate Institute of Injury Prevention and Control, College of Public Health, Taipei Medical University, Taipei, Taiwan; 5 The Innovative and Translational Research Center of Brain Consciousness, Taipei Medical University, Taipei, Taiwan; 6 Department of Psychiatry and Mental Health, Faculty of Health Sciences (FCS-UBI), Cova da Beira University Hospital Center, Covilhã, Portugal

**Keywords:** Biomarkers, Developmental biology, Schizophrenia

## Abstract

Childhood trauma has been linked to schizophrenia, but underlying biological mechanisms remain elusive. This study explored the potential role of plasma oxytocin as a mediator in the relationship between childhood trauma and the psychopathology of schizophrenia. 160 patients with schizophrenia and 80 age- and sex-matched healthy controls were assessed for childhood trauma experiences using the Childhood Trauma Questionnaire and structured interviews. Psychopathology was evaluated using the Positive and Negative Syndrome Scale and plasma oxytocin levels were measured. Results showed that patients with schizophrenia had lower oxytocin levels and higher childhood trauma scores than healthy controls. There was a significant correlation between childhood trauma scores and psychopathology, with plasma oxytocin levels being inversely associated with psychopathology, except for positive symptoms. Hierarchical regression analysis indicated that both childhood trauma scores and plasma oxytocin levels significantly predicted psychopathology. Plasma oxytocin levels partially mediated the relationship between childhood trauma and schizophrenia psychopathology. This study underscores the potential role of oxytocin in bridging the gap between childhood trauma and schizophrenia.

## Introduction

Childhood trauma is recognized as a factor indicating vulnerability to psychotic symptoms and schizophrenia^1,2^. Evidence indicates that patients with schizophrenia are more likely to report a history of childhood adversity or trauma than healthy controls are^3^. Research has consistently identified increased risks of developing psychotic disorders and schizophrenia in the context of various factors among individuals with the experience of child adversity or trauma^4,5^, though establishing direct causality remains complex. Patients with schizophrenia who experienced childhood trauma are typically younger age at schizophrenia onset^6,7^, have worse psychotic symptoms^8,9^, have more severe functional impairment^10,11^, respond to treatment more poorly^12^, and have an even higher risk of suicide^13^ than those who did not experience childhood trauma.

Despite the well-established relationship between childhood trauma and schizophrenia, the mechanisms underlying the association, particularly the biological mechanisms, are poorly understood. Few studies have explored the possible factors mediating this relationship, such as neurotransmitters and hormones. Oxytocin, a hormonal neuropeptide that regulates social cognition, social affiliation, stress, learning and memory^14^, has been reported to have a role in regulating the expression of schizophrenia^15^. Both human and animal studies have explored the role of oxytocin in the development of schizophrenia^16^, particularly its impact on social cognition. Studies examining the endogenous oxytocin levels of patients with schizophrenia have reported mixed findings, with some suggesting that the endogenous oxytocin levels in these patients are lower than those in the healthy population^17^. Oxytocin dysregulation has been demonstrated to be associated with several symptom domains of schizophrenia, particularly negative symptoms and social cognition. A negative correlation between endogenous oxytocin levels and negative symptoms has been reported in numerous studies^18,19^. Oxytocin is critical to the regulation of social cognition in schizophrenia, indicating that patients with higher endogenous oxytocin levels are associated with more effective recognition of facial emotions^20^ and social cues^21^. These findings elucidate the role of oxytocin in the pathophysiology of schizophrenia and have inspired growing research on the therapeutic potential of exogenous oxytocin; some clinical trials have reported encouraging results^22,23^, but the overall findings have been inconsistent. Several factors, such as dosage, route of administration, and individual variations in endogenous oxytocin levels and oxytocin receptor gene, can interfere with treatment efficacy^16,24,25^. Furthermore, it is plausible that individuals with inherently lower endogenous oxytocin levels and oxytocin receptor gene polymorphisms^26,27^, potentially due to factors like childhood trauma, may respond more effectively to intranasal oxytocin treatment. This suggests that intranasal oxytocin could be a viable therapeutic option for patients with schizophrenia, particularly those with a history of childhood trauma. However, further research is required to explore this hypothesis, determine the optimal target groups and treatment course, and gain a more thorough understanding of the mechanisms underpinning the relationship between schizophrenia and oxytocin^28^.

Childhood trauma can have wide-ranging impacts; while some forms involve physical harm, others primarily result in psychological or emotional impacts. These experiences can potentially affect the developing brain, leading to dysregulation in neurotransmitter systems and hormonal production, which may contribute to deficits in behavioral, cognitive, and emotional regulation^29^. Oxytocinergic dysfunction is one of the most studied hormonal disturbances. Most studies have identified an inverse relation between childhood trauma and endogenous oxytocin concentration^30,31^; however, a positive association has also been reported^32,33^. A previous systematic review concluded that reduced oxytocin levels were associated with the history of trauma, supporting the assumption that adversity in early life alters oxytocin homeostasis in the long term^34^. Polymorphism of the oxytocin receptor gene moderates the link between the incidence of childhood abuse and social relationships^35^, implying that childhood trauma may influence the oxytocinergic system through genetic mechanisms. The aforementioned evidence indicates that childhood trauma disrupts the oxytocinergic system, and this disruption may be associated with the progression of schizophrenia. Whether oxytocin mediates the path from childhood trauma to schizophrenia is unconfirmed.

This study explored the relationship between childhood trauma and the clinical symptoms of schizophrenia to investigate the role of plasma oxytocin in this association. New treatment modalities must be developed to address the insufficiency of existing therapies, particularly in alleviating negative symptoms and social cognitive deficits. The identification of key mediators is the first step toward developing new therapeutic agents. In accordance with the literature, we hypothesized the following: (1) patients with schizophrenia are more likely to have childhood trauma experiences and to have experienced more severe trauma compared with healthy controls, (2) patients with schizophrenia have lower plasma oxytocin levels than do healthy controls, (3) a positive correlation exists between the severity of childhood trauma and the severity of schizophrenia psychopathology, and (4) plasma oxytocin levels mediate the relationship between childhood trauma and the severity of schizophrenia psychopathology, with lower plasma oxytocin levels associated with more severe childhood trauma and psychopathology.

## Methods

### Participants and procedures

This cross-sectional study was conducted between August 2020 and April 2022. The study protocol was approved by the Joint Institutional Review Board of Taipei Medical University (Approval No. N202008006, dated August 19, 2020). All procedures contributing to this work comply with the ethical standards of the relevant national and institutional committees on human experimentation and with the Helsinki Declaration of 1975, as revised in 2008. All participants provided written informed consent. Patients with schizophrenia were recruited from the psychiatry outpatient clinic, and healthy controls were enrolled through advertisement. A total of 240 individuals joined the study: 160 patients with schizophrenia and 80 healthy controls matched by age and sex. All participants were aged 20–65 years and capable of providing written informed consent. The *Structured Clinical Interview for DSM-5*^36^ was used as an interview guide by trained psychiatrists to assess diagnoses of any mental disorders for all participants. A patient with schizophrenia was included only if they (i) met the diagnosis criteria for schizophrenia and had been administered a stable dosage of antipsychotic treatment for at least 28 days and (ii) had no current or lifetime mental disorders except for schizophrenia spectrum disorder. Healthy controls were excluded if they or their first-degree relatives had a history of mental disorders. Any participant was excluded if they had a severe neurological disorder, epilepsy, intellectual disability, a neurocognitive disorder, history of substance use disorder, renal disease, or another severe, life-threatening medical condition. They were also excluded if they were pregnant, breastfeeding, or receiving hormonal therapy. No additional treatment was provided to any participant. The patients with schizophrenia received their treatment as usual after recruitment. All assessments were conducted in a private location. Blood tests were performed by nurses on the research team.

### Childhood trauma

All participants were asked to complete the Childhood Trauma Questionnaire—Short Form (CTQ-SF^37^; to screen for and assess the severity of any childhood trauma. The CTQ-SF consists of 28 items and is scored on a 5-point scale. It measures five types of childhood trauma: emotional abuse, emotional neglect, physical abuse, physical neglect, and sexual abuse. The questionnaire has been translated into Chinese, with its reliability confirmed (Cronbach’s α = 0.57 to 0.90; intraclass coefficient = 0.67 to 0.85^38^. Participants who scored at or above the designated moderate exposure cutoff point on each subscale (specifically, ≥10 for physical abuse, ≥13 for emotional abuse, ≥8 for sexual abuse, ≥10 for physical neglect, and ≥15 for emotional neglect) were categorized as individuals with a documented history of childhood trauma exposure^38^. To enhance the validity of these self-reported scores, individual interviews were conducted with all participants. These interviews were carried out by trained psychiatrists following a structured protocol, where they delved deeper into the experiences indicated in the CTQ-SF. Participants were asked to elaborate on their responses, and the psychiatrists probed for specific details and examples of the reported experiences. This process was crucial to ascertain that the reported events met the criteria for childhood trauma as defined in our research context and to distinguish between actual trauma events and other negative, but non-traumatic, childhood experiences. For cross-validation, these detailed clarifications obtained during the interviews were used alongside the questionnaire responses.

### Psychopathology

The Positive and Negative Syndrome Scale (PANSS) was used to evaluate the severity of the psychotic symptoms of patients with schizophrenia. The PANSS is a well-established and widely used scale consisting of 30 items and scored on a 7-point scale; it evaluates the positive, negative, and general psychopathological symptoms of schizophrenia^39^. Studies have demonstrated the robust psychometric properties of this instrument, including the favorable validity of the Chinese version^40^.

### Oxytocin laboratory assessment

Given the challenges of directly measuring central oxytocin levels, we utilized plasma oxytocin levels in our study, informed by studies that identified a positive correlation between central and peripheral concentrations^41^. Phlebotomies were performed in the morning from 8am to 10am. All participants were instructed to abstain from tobacco, caffeine, and analgesics on the day of blood sampling to prevent interference with their plasma oxytocin levels^42,43^. The blood samples were maintained on ice until centrifugation at 3000 rpm for 15 min at 4 °C. Isolated plasma was then divided into 1-mL aliquots and stored at −80 °C immediately until the time of assay. Plasma oxytocin levels were determined using an enzyme immunosorbent assay kit (Catalog number: EKE-051-01, Phoenix Pharmaceuticals, Burlingame, CA, USA) with an oxytocin detection range of 0 to 100 ng/mL. Each plasma sample was assayed twice, and the mean of the two measurements was used in the analysis. We calculated the intra-assay coefficient of variation (CV) by assessing the variability in repeated measurements of the same sample within a single plate during a single run, using two random samples from each plate. The CV for each sample was determined by computing the standard deviation of the first and second results, then dividing this value by the duplicate mean, and finally multiplying by 100. The average CV of all these random samples was taken as the intra-assay CV. For the inter-assay CV, we gauged the variability in measurements of the same sample across different plates. We determined the plate means for the results of two random samples from different plates and then used these values to calculate the overall mean, standard deviation, and CV. The inter-assay and intra-assay coefficients of variation were both less than 5%, and no significant cross reactivity or interference between oxytocin and analogs was observed.

### Covariates

Demographic characteristics, disease-specific variables, and cognitive function were added to the analysis as covariates, as they potentially influence the relationships between childhood trauma, oxytocin, and psychopathology. The inclusion of age and sex as confounders is based on their known influence on the onset, progression, and psychopathology of schizophrenia^44,45^, as well as their potential impact on plasma oxytocin levels^46^. Regarding disease-specific variables, the age of schizophrenia onset and illness duration were considered colliders of childhood trauma and plasma oxytocin levels^47,48^. Antipsychotics were recorded and converted into chlorpromazine-equivalent doses^49^, considering their established relevance in schizophrenia psychopathology and plasma oxytocin levels^15,50^. The Mini-Mental State Examination (MMSE) scores and years of education were included as confounders due to their potential impact on cognitive function, which may confound the relationship between childhood trauma and psychopathology^51,52^. The MMSE^53^ is a 21-item instrument with scores ranging from 0 to 30 to assess the following domains: orientation, registration and recall, attention and calculation, language, repetition, and the ability to follow written and verbal instructions. The decision to incorporate these covariates was informed by both empirical evidence and theoretical considerations, aiming to elucidate the complex interplay between these variables and the psychopathology of schizophrenia.

### Statistical analysis

All collected data were transcribed in Microsoft Excel and then transferred to SPSS Statistics version 26.0 (IBM, Armonk, NY, USA) for coding and analysis. The normality of distributions was determined using the Kolmogorov–Smirnov test. The demographic characteristics, disease-specific variables, MMSE scores, plasma oxytocin levels, CTQ-SF scores, and PANSS scores are expressed as the mean (*M*) with standard deviation (*SD*). Independent sample *t*-tests were employed to compare continuous variables between the schizophrenia group and healthy controls, with Cohen’s d used to quantify effect size. The variables under comparison included demographic characteristics (age, sex, years of education, and MMSE score); plasma oxytocin levels; scores for each component of the CTQ-SF and the number of types of childhood trauma. Pearson’s chi-square test was applied to assess the categorical variable, namely, the prevalence of various types of childhood trauma among healthy controls and patients with schizophrenia, with effect sizes determined using Cramér’s V (φ_c_﻿). A series of one-way analyses of variance (ANOVAs) were employed to investigate the relationship between various types of childhood trauma and the severity of schizophrenia symptoms, as measured by the PANSS. Eta-squared (η^2^) was used to quantify the effect size for each ANOVA performed, providing a measure of the strength of the associations.

Pearson’s correlation coefficients were calculated to examine the correlations between psychopathology (measured by the PANSS total score, positive scale, negative scale, and general psychopathology scale) and other continuous variables, including plasma oxytocin levels, CTQ-SF score, demographic characteristics (sex, age, and years of education), disease-specific variables (age of schizophrenia onset, duration of illness, and antipsychotic dose), and MMSE score. Hierarchical regression analysis was used to investigate whether childhood trauma and plasma oxytocin levels accounted for unique variance in the psychopathology (measured by PANSS total score) of schizophrenia beyond that explained by other covariates (i.e., sex, age, years of education, age of schizophrenia onset, antipsychotic dose, and MMSE score). The covariates were included in the regression analysis model hierarchically in accordance with a time series. Finally, to explore the potential association between plasma oxytocin levels and the psychopathology (measured by PANSS total score) of schizophrenia, and how this might relate to childhood trauma, a mediation analysis was conducted using SPSS macro-PROCESS version 4.1 (model 4)^54^; after the data had been bias-corrected and percentile-method bootstrapped, with the data resampled 5,000 times. Exploratory analyses were performed to determine the best-fit model for the mediation analysis. The covariates incorporated into the mediation analysis were sex, age, years of education, age of schizophrenia onset, antipsychotic dose, and MMSE score. As the issue of multiple comparisons was present in this study, Bonferroni correction was applied to adjust the significance level. All probability values are reported at the two-tailed level for statistical significance at *p* < 0.05.

## Results

### Demographic characteristics, plasma oxytocin levels, childhood trauma, and psychopathology

**Fig. 1:**
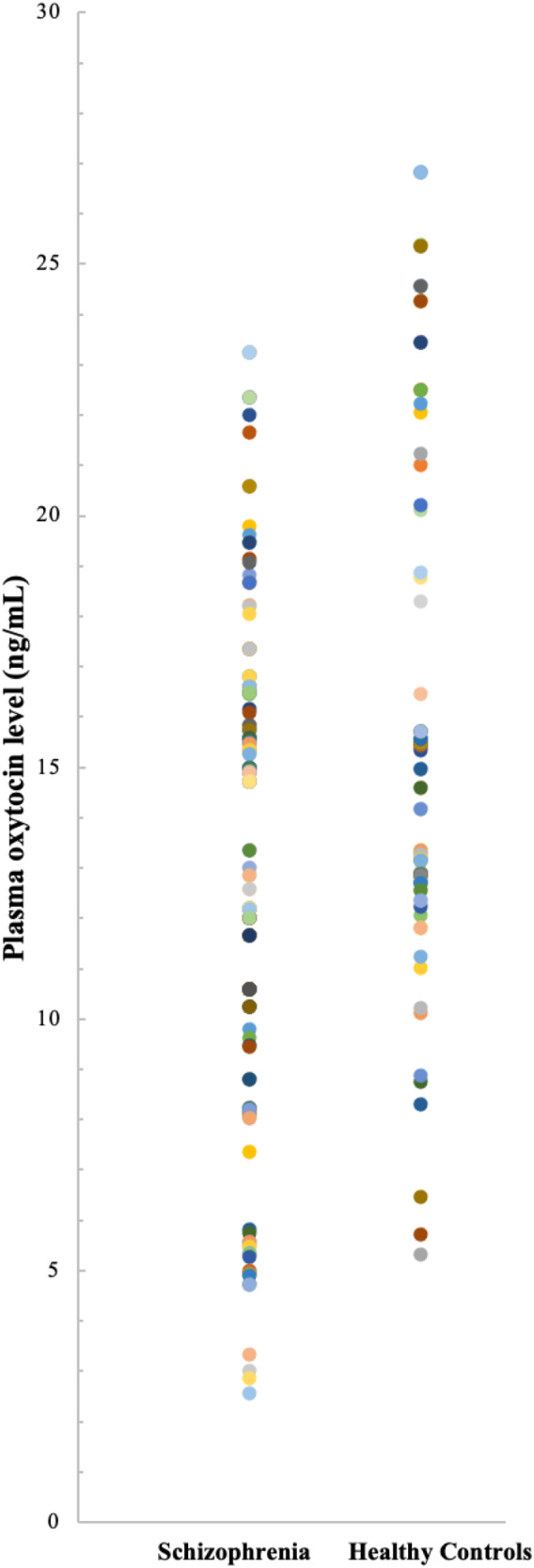
Distribution of plasma oxytocin levels between patients with schizophrenia and healthy controls.

The demographic characteristics and childhood trauma of the patients with schizophrenia and healthy controls are presented in Table 1. No significant differences between the two groups in terms of age and sex, but there was a significant difference in the years of education, with the schizophrenia group having lower years of education compared to the healthy group (*t* = −2.093, *p* = 0.038). All participants underwent the MMSE evaluation and were found to have normal cognitive function, with no intergroup differences being discovered. Figure 1 illustrates the distribution of plasma oxytocin levels in patients with schizophrenia compared to healthy controls. In comparison with the healthy controls, the patients with schizophrenia had significantly lower plasma oxytocin levels (*t* = −5.543, *p* < 0.001). The total scores in the CTQ-SF, as well as the scores in all the subscales, were higher for the patients with schizophrenia than for the healthy controls (*p* < 0.001). Comparison of the prevalence of different trauma types between healthy controls and patients with schizophrenia revealed a higher prevalence of all types of childhood trauma in the latter group: physical abuse (*p* < 0.001), emotional abuse (*p* = 0.004), sexual abuse (*p* = 0.008), physical neglect (*p* = 0.012), emotional neglect (*p* = 0.015), as well as a greater number of trauma types (*p* < 0.001). Table 1 provides information about the psychopathology and disease-specific variables of the patients with schizophrenia.

**Table 1:** Demographic characteristics of patients with schizophrenia and healthy controls.

	Schizophrenia *n* = 160		Healthy Controls *n* = 80		Significance		
	*M*	*SD*	*M*	*SD*	*t/χ²*	*p*	*d/φ*_*c*_
Age	42.40	9.40	43.45	9.99	−0.783	0.435	−0.109
Sex (male/female)	96/64		46/34			0.710	
Education years	10.58	3.00	11.46	3.15	−2.093	0.038	−0.288
MMSE score	29.23	0.97	29.50	1.00	−1.976	0.050	−0.275
Plasma oxytocin levels (ng/mL)	14.34	3.07	17.26	4.49	−5.543	< 0.001	−0.810
*CTQ-SF score*							
Total score	60.59	14.18	38.00	4.32	13.917	< 0.001	2.155
Physical abuse	13.59	5.00	7.16	1.66	11.175	< 0.001^a^	1.532
Emotional abuse	12.52	7.37	6.96	1.24	6.693	< 0.001^a^	0.917
Sexual abuse	6.98	3.25	5.19	0.55	4.881	< 0 .001^a^	0.669
Physical neglect	12.38	5.05	10.50	1.40	3.266	0.006^a^	0.447
Emotional neglect	15.19	6.81	8.19	2.04	9.000	< 0.001^a^	1.230
*Positive for childhood trauma (n)*							
Physical abuse		118		32	25.920	< 0.001	0.329
Emotional abuse		71		20	8.505	0.004	0.188
Sexual abuse		39		8	6.998	0.008	0.171
Physical neglect		103		38	6.267	0.012	0.162
Emotional neglect		70		22	5.958	0.015	0.158
Number of childhood trauma types	2.51	1.90	1.50	1.35	4.725	< 0.001	0.610
Age of schizophrenia onset	24.21	8.28					
Duration of illness	18.19	10.65					
Antipsychotic dose (CPZ equiv. in mg)	393.43	323.90					
*PANSS score*							
Total score	76.22	17.22					
Positive symptoms	18.08	6.98					
Negative symptoms	19.91	8.05					
General psychopathology	33.99	7.54					

*MMSE* Mini-Mental Status Examination, *CTQ-SF* Childhood Trauma Questionnaire—Short Form, *CPZ equiv.* chlorpromazine equivalent doses, *PANSS* Positive and Negative Syndrome Scale.

^a^Bonferroni-corrected *p* value.

Table 2 revealed significant associations between childhood trauma and PANSS scores among patients with schizophrenia. Patients who reported experiencing any form of childhood trauma—and who scored at or above the designated moderate exposure cutoff points on each subscale of the CTQ-SF, thus categorized as individuals with a documented history of childhood trauma exposure—consistently had higher PANSS total scores. This indicates more severe psychopathology compared to those without such childhood trauma history. Specifically, emotional abuse and emotional neglect were associated with the highest increases in PANSS total scores, as well as in scores for negative symptoms and general psychopathology. The effect sizes, as indicated by η^2^, ranged from moderate to large across different types of childhood trauma, with emotional neglect showing the most substantial impact on all measured aspects of the PANSS subdomains.

**Table 2:** Comparison of psychopathology among patients with schizophrenia based on various childhood trauma types.

	PANSS							
	Total score		Positive symptoms		Negative symptoms		General psychopathology	
	*M*	*SD*	*M*	*SD*	*M*	*SD*	*M*	*SD*
*Physical abuse*								
Yes (*n* = 118)	81.50	14.79	19.07	6.51	21.89	7.82	36.08	6.83
No (*n* = 42)	61.38	14.84	15.31	7.55	14.33	5.79	28.12	6.27
	*F*(1, 158) = 57.219; *p* < 0.001; η^2^ = 0.266		*F*(1, 158) = 9.466; *p* = 0.002; η^2^ = 0.057		*F*(1, 158) = 32.750; *p* < 0.001; η^2^ = 0.172		*F*(1, 158) = 43.914; *p* < 0.001; η^2^ = 0.217	
*Emotional abuse*								
Yes (*n* = 71)	89.77	8.98	20.73	6.32	24.59	7.78	39.51	5.35
No (*n* = 89)	65.40	14.32	15.97	6.78	16.17	6.09	29.60	5.98
	*F*(1, 158) = 156.345; *p* < 0.001; η^2^ = 0.497		*F*(1, 158) = 20.715; *p* < 0.001; η^2^ = 0.116		*F*(1, 158) = 59.031; *p* < 0.001; η^2^ = 0.272		*F*(1, 158) = 118.865; *p* < 0.001; η^2^ = 0.429	
*Sexual abuse*								
Yes (*n* = 39)	89.05	9.69	20.41	6.90	24.67	7.98	38.85	5.16
No (*n* = 121)	72.08	17.10	17.33	6.86	18.37	7.48	32.43	7.53
	*F*(1, 158) = 34.701; *p* < 0.001; η^2^ = 0.180		*F*(1, 158) = 5.924; *p* = 0.016; η^2^ = 0.036		*F*(1, 158) = 20.218; *p* < 0.001; η^2^ = 0.113		*F*(1, 158) = 24.525; *p* < 0.001; η^2^ = 0.134	
*Physical neglect*								
Yes (*n* = 103)	82.54	15.76	19.14	6.88	22.23	8.47	36.63	7.14
No (*n* = 57)	64.79	13.54	16.18	6.80	15.70	5.04	29.23	5.71
	*F*(1, 158) = 51.344; *p* < 0.001; η^2^ = 0.245		*F*(1, 158) = 6.850; *p* = 0.010; η^2^ = 0.042		*F*(1, 158) = 28.308; *p* < 0.001; η^2^ = 0.152		*F*(1, 158) = 45.223; *p* < 0.001; η^2^ = 0.223	
*Emotional neglect*								
Yes (*n* = 70)	90.16	9.25	20.57	6.55	25.07	7.99	39.54	5.37
No (*n* = 90)	65.38	13.85	16.14	6.71	15.89	5.38	29.68	6.01
	*F*(1, 158) = 166.212; *p* < 0.001; η^2^ = 0.513		*F*(1, 158) = 17.497; *p* < 0.001; η^2^ = 0.100		*F*(1, 158) = 75.137; *p* < 0.001; η^2^ = 0.322		*F*(1, 158) = 116.320; *p* < 0.001; η^2^ = 0.424	

### Correlations between psychopathology and other variables

Correlation analysis within the schizophrenia cohort, as detailed in Table 3, indicated a significant correlation between the total CTQ-SF score and the total PANSS score (*r* = 0.699, *p* < 0.001). Additionally, all scores for the CTQ-SF subscales were significantly correlated with the total PANSS score and the scores for its subdomains scores, except for positive symptoms. Besides, the higher number of childhood trauma types experienced, the greater severity of schizophrenia psychopathology, measured by the total PANSS score (*r* = 0.738, *p* < 0.001) and the scores for its subdomains, namely positive symptoms (*r* = 0.843, *p* < 0.001), negative symptoms (*r* = 0.951, *p* < 0.001), and general psychopathology (*r* = 0.845, *p* < 0.001). Plasma oxytocin levels were inversely correlated with the total PANSS score (*r* = −0.688, *p* < 0.001) and the scores for its subdomains, except for positive symptoms.

**Table 3:** Pearson’s correlation coefficients between childhood trauma and psychopathology in patients with schizophrenia.

	PANSS											
	Total score			Positive symptoms			Negative symptoms			General psychopathology		
	*r*	*r*^*2*^	*p*^a^	*r*	*r*^*2*^	*p*^a^	*r*	*r*^*2*^	*p*^a^	*r*	*r*^*2*^	*p*^a^
*CTQ-SF score*												
Total score	0.699	0.489	< 0.001	0.253	0.064	0.069	0.603	0.364	< 0.001	0.639	0.408	< 0.001
Physical abuse	0.654	0.428	< 0.001	0.263	0.069	0.054	0.562	0.316	< 0.001	0.581	0.338	< 0.001
Emotional abuse	0.667	0.445	< 0.001	0.230	0.053	0.192	0.559	0.312	< 0.001	0.630	0.397	< 0.001
Sexual abuse	0.302	0.091	0.006	0.015	0.000	1.000	0.357	0.127	< 0.001	0.261	0.068	0.044
Physical neglect	0.588	0.346	< 0.001	0.212	0.045	0.399	0.518	0.268	< 0.001	0.540	0.292	< 0.001
Emotional neglect	0.705	0.497	< 0.001	0.282	0.080	0.057	0.582	0.339	< 0.001	0.643	0.413	< 0.001
Number of childhood trauma types	0.738	0.544	< 0.001	0.843	0.710	< 0.001	0.951	0.904	< 0.001	0.845	0.714	< 0.001
Sex	−0.066	0.004	1.000	−0.063	0.004	1.000	−0.005	0.000	1.000	−0.093	0.009	0.976
Age	−0.085	0.007	1.000	0.029	0.000	1.000	−0.097	0.009	0.888	−0.104	0.011	0.768
Education years	−0.136	0.018	0.348	−0.032	0.001	1.000	−0.107	0.011	0.708	−0.133	0.018	0.376
Age of schizophrenia onset	−0.160	0.026	0.172	0.009	0.000	1.000	−0.203	0.041	0.040	−0.162	0.026	0.164
Duration of illness	0.049	0.002	1.000	0.018	0.000	1.000	0.072	0.005	1.000	0.034	0.001	1.000
Antipsychotic dose	0.080	0.006	1.000	0.100	0.010	0.836	0.006	0.000	1.000	0.049	0.002	1.000
MMSE score	−0.121	0.015	0.512	0.112	0.013	0.636	−0.223	0.050	0.020	−0.147	0.022	0.256
Plasma oxytocin levels	−0.688	0.473	< 0.001	−0.161	0.026	0.168	−0.646	0.417	< 0 .001	−0.647	0.419	< 0.001

*MMSE* Mini-Mental Status Examination, *CTQ-SF* Childhood Trauma Questionnaire—Short Form, *PANSS* Positive and Negative Syndrome Scale.

^a^Bonferroni-corrected *p* value.

Age of schizophrenia onset was discovered to have a negative association with the total PANSS score, negative symptoms, and general psychopathology; however, after applying the Bonferroni correction, this association remained significant only for negative symptoms (*r* = −0.203, *p* = 0.040), indicating that earlier onset is associated with more severe negative symptoms. Regarding the other variables, the correlations were nonsignificant except for a negative association between the MMSE score and negative symptoms (*r* = −0.223, *p* = 0.020).

### Hierarchical regression analysis of predictors of psychopathology

The results of the hierarchical regression analysis are provided in Table 4. The variables were included in succeeding steps: (a) model 1 predicted psychopathology from only sex and age, (b) CTQ-SF score was added for model 2, (c) educational years was added for model 3, (d) age of schizophrenia onset was added for model 4, (e) antipsychotic dose and MMSE score was added for model 5, and (f) plasma oxytocin levels were added for model 6. The result of model 2 revealed that the CTQ-SF score served as a significant predictor of psychopathology, explaining 47.7% of the variation (Δ*R*^*2*^ = 0.477, *p* < 0.001). The result of model 6 demonstrated that oxytocin levels accounted for an additional 6.1% change in the prediction of psychopathology (Δ*R*^*2*^ = 0.052, *p* < 0.001). Additional hierarchical regression analyses predicting the PANSS subdomains scores were presented in Supplementary Table S1.

**Table 4:** Hierarchical regression analysis of predictors of psychopathology (measured by PANSS total score) in patients with schizophrenia.

	*R*	*R*^*2*^	*∆R*^*2*^	*p*	*B*	*SE B*	β	*t*	*p*
Model 1	0.116	0.013		0.344					
Sex					−2.795	2.806	−0.080	−0.996	0.321
Age					−0.177	0.147	−0.096	−1.205	0.230
Model 2	0.701	0.491	0.477	< 0.001					
Sex					−1.683	2.025	−0.048	−0.831	0.407
Age					−0.028	0.106	−0.015	−0.261	0.795
CTQ-SF score					0.496	0.041	0.696	12.091	< 0.001
Model 3	0.707	0.499	0.009	0.102					
Sex					−1.627	2.014	−0.046	−0.808	0.421
Age					−0.058	0.107	−0.032	−0.538	0.591
CTQ-SF score					0.490	0.041	0.688	11.979	< 0.001
Education years					−0.547	0.332	−0.095	−1.645	0.102
Model 4	0.711	0.506	0.007	0.153					
Sex					−2.120	2.036	−0.061	−1.041	0.299
Age					−0.022	0.110	−0.012	−0.204	0.839
CTQ-SF score					0.482	0.041	0.676	11.700	< 0.001
Education years					−0.610	0.334	−0.106	−1.825	0.070
Age of schizophrenia onset					−0.182	0.126	−0.087	−1.436	0.153
Model 5	0.719	0.516	0.010	0.198					
Sex					−2.077	2.037	−0.059	−1.020	0.310
Age					−0.016	0.110	−0.008	−0.142	0.888
CTQ-SF score					0.477	0.041	0.670	11.582	< 0.001
Education years					−0.652	0.336	−0.113	−1.939	0.054
Age of schizophrenia onset					−0.213	0.129	−0.102	−1.653	0.100
Antipsychotic dose					0.005	0.003	0.101	1.745	0.083
MMSE score					−0.536	1.030	−0.030	−0.521	0.603
Model 6	0.754	0.569	0.052	< 0.001					
Sex					−2.015	1.931	−0.057	−1.043	0.298
Age					−0.030	0.104	−0.016	−0.290	0.772
CTQ-SF score					0.294	0.058	0.413	5.078	< 0.001
Education years					−0.351	0.326	−0.061	−1.074	0.285
Age of schizophrenia onset					−0.174	0.122	−0.083	−1.418	0.158
Antipsychotic dose					0.005	0.003	0.095	1.732	0.085
MMSE score					0.244	0.993	0.014	0.246	0.806
Plasma oxytocin levels					−62.896	14.733	−0.359	−4.269	< 0.001

*MMSE* Mini-Mental Status Examination, *CTQ-SF* Childhood Trauma Questionnaire—Short Form, *PANSS* Positive and Negative Syndrome Scale.

### Mediation effect of plasma oxytocin

Mediation analysis results, presented in Table 5, demonstrated a significant regression coefficient between childhood trauma and plasma oxytocin levels (*p* < 0.001), and between plasma oxytocin levels and the psychopathology of schizophrenia (*p* < 0.001). The bootstrapped unstandardized indirect effect was significant (β = 0.183, *SE* = 0.044, 95% confidence interval [CI] [0.102, 0.272]), indicating that plasma oxytocin levels partially mediate the effect of childhood trauma on the schizophrenia psychopathology. The regression coefficients remained robust when controlling for covariates presented in the study such as age, sex, years of education, MMSE score, age of schizophrenia onset, and antipsychotic dosage. The overall mediation model was significant (*R*^2^ = 0.517, *F* = 23.195, *p* < 0.001). Furthermore, additional analyses were conducted and presented in Supplementary Tables S2 and S3, where the correlations between PANSS scores and the number of childhood trauma types were examined, as well as their implications in hierarchical regression and mediation analysis. These analyses corroborated the initial findings that using total CTQ-SF scores, underscoring the robustness of the results. All exploratory analyses conducted to determine the best-fit model for the mediation analysis are presented in Supplementary Table S4.

**Table 5:** Mediation effect of plasma oxytocin levels on the relationship between childhood trauma and psychopathology (measured by PANSS total score) in patients with schizophrenia.


Model summary						
	*R*	*R*^*2*^	*MSE*	*F*	*p*	
Model 4	0.719	0.517	150.014	23.195	< 0.001	
Covariates: sex, age, year of education, MMSE score, age of schizophrenia onset, antipsychotic dose.						

*Mediation Estimates*						
Effect	Estimate	*SE*	95% CI	% Mediation		
Indirect	0.183	0.044	[0.102, 0.272]	38.36		
Direct	0.294	0.058	[0.180, 0.409]	61.64		
Total	0.477	0.041	[0.400, 0.559]	100.00		

*Path Estimates*						
			Estimate	*SE*	*t*	*p*^a^
CTQ-SF score	→	Oxytocin levels	−0.003	0.001	−13.522	< 0.001
Oxytocin levels	→	PANSS total score	−62.896	14.733	−4.269	< 0.001
CTQ-SF score	→	PANSS total score	0.295	0.058	5.079	< 0.001

*MMSE* Mini-Mental Status Examination, *CTQ-SF* Childhood Trauma Questionnaire—Short Form, *PANSS* Positive and Negative Syndrome Scale.

^a^Bonferroni-corrected *p* value.

## Discussion

The results of this study indicated that any childhood trauma experienced by the participants was more severe on average in the patients with schizophrenia than in the healthy controls, as reflected in the significant difference between CTQ-SF scores and in the prevalence of positive responses for various types of childhood trauma. The patients with schizophrenia also had lower oxytocin levels, which is consistent with our hypothesis and supports the idea that oxytocinergic system dysfunction is associated with schizophrenia. We further investigated the link between childhood trauma and psychopathology severity in patients with schizophrenia, determining a positive correlation between these two variables; plasma oxytocin levels were inversely correlated with both factors. To provide new insights into these associations, we examined the role of oxytocin through mediation analysis. After controlling for covariates, oxytocin was found to exert a partial mediation effect on the relationship between childhood trauma and the psychopathology of schizophrenia.

Our findings align with the existing literature indicating that patients with schizophrenia experience more severe childhood trauma, which is substantiated by both the higher CTQ-SF total scores and the increased prevalence of all trauma types in this population, with the existence of potential threshold and dose–response effects^55–58^. This observed gradation in childhood trauma severity and its association with the spectrum of schizophrenia symptoms highlight the intricacy of trauma’s impact on the disorder. Our findings reveal that a categorical approach to assessing childhood trauma—classifying individuals based on whether their experiences meet a certain threshold of severity—aligns with heightened positive symptoms in schizophrenia. In contrast, our correlation analysis did not show a direct relationship between the continuous severity of childhood trauma and positive symptoms. Our results may suggest that while childhood trauma, in general, is associated with an exacerbation of psychopathology, the specific relationship with positive symptoms may become more pronounced only after surpassing a certain threshold of trauma severity. This nuanced effect is in line with the notion that various trauma subtypes may influence the development of schizophrenia to differing extents, highlighting the potential for threshold effects in the trauma-psychopathology nexus, as supported by a meta-analysis indicating that all trauma subtypes may confer a substantial risk of psychosis^4^. The significant correlation between the number of childhood trauma types and the severity of psychopathology in our schizophrenia cohort further reinforces the concept of a dose-response relationship, where a greater number of trauma experiences correlates with more severe symptoms of the disorder^59,60^. Such severity may trigger a series of neurobiological changes, including HPA axis dysregulation, genetic vulnerabilities, and epigenetic modifications, contributing to the altered brain structure and function observed in schizophrenia^61^.

The interplay between childhood trauma and schizophrenia is multifaceted, with research suggesting that the dissociative states stemming from adversity could amplify cognitive deficits. Such deficits may blur the distinction between internal thoughts and external reality, potentially leading to hallucinations. These hallucinations may manifest as a variation of posttraumatic intrusive memories^62^ or emerge from errors in source monitoring^63,64^. Notably, auditory verbal hallucinations have been linked to these cognitive challenges in discerning internal from external auditory information, a difficulty that may be exacerbated by dissociative states induced by childhood trauma^65–67^, and potentially related to oxytocinergic system dysfunctions^68^. Furthermore, delusions, particularly those connected to trauma-induced negative beliefs, can arise from a compromised ability to form secure attachments due to childhood negligence, fostering distrust and paranoia. This attachment failure and the ensuing paranoia may be moderated by genetic factors, including polymorphisms in the oxytocin receptor gene^69,70^. Moreover, attachment style has been implicated in the development of negative symptoms^71,72^, with poor attachment possibly leading to interpersonal dysfunction. This dysfunction has been hypothesized as an adaptive deactivation of the attachment system in response to the fear of rejection or threat^73^, further complicating the clinical presentation of schizophrenia. These findings are consistent with evidence linking various trauma subtypes to schizophrenia, particularly to negative symptoms and general psychopathology. However, our results indicate that the association with positive symptoms is less pronounced, a finding that diverges from some studies emphasizing the childhood trauma-positive symptom connection^58,74^, yet aligns with others that have found a stronger link between childhood trauma, especially neglectful trauma, and negative symptoms^75–77^.

Building on this, the nuances in the relationships between specific childhood trauma types and schizophrenia symptoms become evident. Although sexual abuse was found to have a weaker association with schizophrenia in our study, it is essential to consider the broader context of research. For instance, the literature presents mixed evidence on sexual abuse’s connection with schizophrenia, with some studies indicating a particular association with positive symptoms, while others do not find a strong link to negative symptoms^78–81^. This variability suggests that positive and negative symptoms may emerge through distinct pathways related to the impact of sexual abuse. Furthermore, the potential for underreporting of sexual abuse due to stigma, guilt, or embarrassment^82^, as well as the dissociative amnesia^83^, introduces additional challenges in assessing the true strength of these associations. Such complexities underscore the need for a careful examination of how different childhood trauma experiences contribute to the heterogeneity of schizophrenia’s symptomatology.

Our study contributes to the growing body of literature that posits the oxytocinergic system as a potential mediator in the pathway from childhood trauma to the development of schizophrenia. This study introduces a potential new explanation for the trajectory from childhood trauma to schizophrenia, with oxytocin possibly playing a significant role. However, the intricate mechanisms through which childhood trauma disrupts the oxytocinergic system are still an area of active exploration. The disruption of early attachment processes, which are intricately linked to oxytocin regulation, appears to be a contributing factor to the emergence of negative symptoms and general psychopathology^84–86^. The resulting alteration in oxytocin levels might influence key neural pathways, including those involving oxytocin’s interaction with dopaminergic pathways, regulation of the amygdala, and adjustment of social information processing^87^, which provide potential explanations for our observations. Indeed, oxytocin dysregulation could exacerbate social cognitive deficits, potentially leading to enhanced paranoia, social withdrawal, and comorbid affective disorders, as evidenced by our findings and supported by recent studies^27,88,89^. This proposition aligns with our observation that oxytocin levels are associated with various symptom domains of schizophrenia.

Furthermore, initial molecular insights suggest that the impact of childhood trauma may extend to the genetic regulation of oxytocin production and receptor expression^90^, potentially via epigenetic modifications like DNA methylation^91^ or single nucleotide polymorphisms^92^, both of which can induce stress-related pathology^93^. This genetic vulnerability, compounded by adverse environmental exposures, may contribute to a ‘gene × environment’ interplay that underpins the schizophrenia phenotype. While oxytocin’s role in this complex interplay is significant, it is likely not the sole mediator. Our study echoes the broader schizophrenia research that implicates factors such as chronic stress and systemic inflammation as additional contributing elements to the psychopathology^61,94^. As such, our findings underscore the need for a multifactorial approach to understand the full scope of schizophrenia’s etiology and pathophysiology, considering both neurobiological and environmental influences.

To our knowledge, this is the first study to explore oxytocin’s mediating role in the relationship between childhood trauma and schizophrenia. However, the findings must be interpreted cautiously due to the study’s limitations. The retrospective nature of the CTQ-SF raises concerns about recall bias and potential underreporting of childhood trauma^95^, although prior research supports the validity of retrospective reporting^96^. We attempted to minimize bias by conducting interviews to clarify and validate participant responses. Nevertheless, future research employing mediation analyses and a prospective design would be invaluable in confirming the mediating role of oxytocin. Oxytocin levels are subject to various influences, such as stress, inflammation, circadian rhythm, nicotine use^97^, and reproductive status^46^, which could act as uncontrolled confounders. We have attempted to control for some of these by excluding nicotine use, pregnancy, breastfeeding, hormonal therapy, and menstruation from our participant criteria. We requested that participants refrain from tobacco use on the day of blood sampling. We also ensured that blood sampling did not occur during menstruation. Additionally, the potential influence of antipsychotic medication on oxytocin levels should be considered. Furthermore, while we standardized antipsychotic dosages to chlorpromazine-equivalent doses, the lack of differentiation between antipsychotic types is a limitation given their distinct effects on oxytocin levels. Subsequent studies should differentiate between antipsychotic classes to better understand their impact on oxytocin and schizophrenia. Furthermore, assessing oxytocin levels both before and after antipsychotic treatment would be instrumental in better understanding the dynamic between medication and oxytocin regulation in schizophrenia. Moreover, while peripheral oxytocin levels correlate with central levels^41^, they are not a perfect substitute. Direct measurements of central oxytocin remain impractical, posing a challenge for accurately assessing its role in schizophrenia. Additionally, our study specifically included participants with schizophrenia who had been on stable medication for at least 28 days to minimize the confounding effects of fluctuating medication levels on oxytocin levels and psychopathology. While this approach was aimed at strengthening the internal validity of our findings, it may also have inadvertently introduced a selection bias. By selecting more clinically stable participants, our findings may not fully represent the broader schizophrenia population, particularly those not on consistent medication regimens or those with additional comorbidities. This could potentially limit the generalizability of our results to all cases of schizophrenia. The cross-sectional design also means we cannot establish causal links or account for variations in trauma timing^98^. Despite these limitations, we constructed mediation models based on the assumption of oxytocin’s stability over time, aiming to elucidate its relationship with psychopathology of schizophrenia. Future longitudinal studies are necessary to confirm these relationships over time. Our study underscores the significance of oxytocin in understanding the biological impact of childhood trauma on schizophrenia, highlighting the need for further investigation into the genetic factors that may affect oxytocin regulation following trauma. Identifying specific genetic variants linked to schizophrenia susceptibility could provide valuable insights for therapeutic interventions. Future clinical trials might consider including individuals with a history of childhood trauma or those with suboptimal oxytocin levels to discern who might benefit most from oxytocin supplementation.

## Conclusion

Childhood trauma is recognized a major risk factor in the development of schizophrenia. In this cross-sectional study, we delved into the underlying mechanisms, examining the role of oxytocin in mediating the effects of childhood trauma on the development of schizophrenia. Consistent with prior research, our findings confirm a positive correlation between childhood trauma and the severity of schizophrenia, as well as an inverse correlation between oxytocin levels and these variables. Significantly, our findings suggest that oxytocin partially mediates the relationship between childhood trauma and the clinical manifestations of schizophrenia, indicating the childhood trauma may potentially led to oxytocin dysregulation, which in turn could increase the clinical severity of schizophrenia. These insights reinforce the crucial need for preventive measures, early recognition, and targeted interventions in schizophrenia, particularly in individuals with a history of childhood trauma. The potential of oxytocin as a therapeutic avenue for alleviating symptoms offers a promising direction for future clinical research and treatment strategies in schizophrenia.

### Supplementary information

Supplements

## Data Availability

Supplementary information is available for this paper. The datasets generated during and/or analyzed during this study are available from the corresponding author upon reasonable request.
