# Supplementary material for: Linking childhood trauma to the psychopathology of schizophrenia: the role of oxytocin
Source: Schizophrenia (Heidelb). 2024 Feb 22;10(1):24. doi: 10.1038/s41537-024-00433-9 (PMC10883944; doi:10.1038/s41537-024-00433-9)
Supplement: Supplementary file 1 — Supplements [file 41537_2024_433_MOESM1_ESM.docx]

Table 1. Demographic, clinical data, and neuropsychological background tests scores of patients with schizophrenia and normal

control

**Supplementary Table Captions**

Table S1. Hierarchical regression analysis of predictors of psychopathology (measured by PANSS subdomains scores) in patients with schizophrenia.

Table S2. Hierarchical regression analysis of predictors of psychopathology (measured by PANSS total score) in patients with schizophrenia, using number of childhood trauma types.

Table S3. Mediation effect of plasma oxytocin level on the relationship between childhood trauma and psychopathology (measured by PANSS total score) in patients with schizophrenia, using number of childhood trauma types.

Table S4. Summary of theoretical exploratory analyses for mediation models.

**Table S1.** Hierarchical regression analysis of predictors of psychopathology (measured by PANSS subdomains scores) in patients with schizophrenia.

| **PANSS Positive** | *R* | *R^2^* | *∆R^2^* | *p* | *B* | *SE B* | β | *t* | *p* |
| --- | --- | --- | --- | --- | --- | --- | --- | --- | --- |
| **Model 6** | .313 | .098 | .050 | .786 |  |  |  |  |  |
| Sex |  |  |  |  | −.400 | 1.131 | −.028 | −.354 | .724 |
| Age |  |  |  |  | .038 | .061 | .051 | .627 | .531 |
| CTQ-SF score |  |  |  |  | .085 | .034 | .294 | 2.500 | **.013** |
| Education years |  |  |  |  | −.073 | .191 | −.031 | −.379 | .705 |
| Age of schizophrenia onset |  |  |  |  | −.009 | .072 | −.011 | −.129 | .897 |
| Antipsychotic dose |  |  |  |  | .002 | .002 | .093 | 1.181 | .239 |
| MMSE score |  |  |  |  | .966 | .582 | .134 | 1.660 | .099 |
| Plasma oxytocin level |  |  |  |  | 2.343 | 8.632 | .033 | .271 | .786 |

| **PANSS Negative** | *R* | *R^2^* | *∆R^2^* | *p* | *B* | *SE B* | β | *t* | *p* |
| --- | --- | --- | --- | --- | --- | --- | --- | --- | --- |
| **Model 6** | .686 | .470 | .442 | **< .001** |  |  |  |  |  |
| Sex |  |  |  |  | −.332 | 1.000 | −.020 | −.332 | .741 |
| Age |  |  |  |  | −.020 | .054 | −.024 | −.377 | .707 |
| CTQ-SF score |  |  |  |  | .091 | .030 | .274 | 3.036 | **.003** |
| Education years |  |  |  |  | −.066 | .169 | −.025 | −.391 | .696 |
| Age of schizophrenia onset |  |  |  |  | −.098 | .063 | −.101 | −1.543 | .125 |
| Antipsychotic dose |  |  |  |  | .001 | .002 | .031 | .505 | .614 |
| MMSE score |  |  |  |  | −.731 | .514 | −.088 | −1.422 | .157 |
| Plasma oxytocin level |  |  |  |  | −32.956 | 7.630 | −.402 | −4.319 | **< .001** |

| **PANSS General** | *R* | *R^2^* | *∆R^2^* | *p* | *B* | *SE B* | β | *t* | *p* |
| --- | --- | --- | --- | --- | --- | --- | --- | --- | --- |
| **Model 6** | .703 | .494 | .467 | **< .001** |  |  |  |  |  |
| Sex |  |  |  |  | −1.475 | .915 | −.096 | −1.612 | .109 |
| Age |  |  |  |  | −.038 | .049 | −.047 | −.767 | .445 |
| CTQ-SF score |  |  |  |  | .108 | .027 | .346 | 3.931 | **< .001** |
| Education years |  |  |  |  | −.152 | .155 | −.060 | −.983 | .327 |
| Age of schizophrenia onset |  |  |  |  | −.077 | .058 | −.084 | −1.318 | .190 |
| Antipsychotic dose |  |  |  |  | .001 | .001 | .063 | 1.068 | .287 |
| MMSE score |  |  |  |  | −.168 | .471 | −.022 | −.357 | .722 |
| Plasma oxytocin level |  |  |  |  | −27.414 | 6.983 | −.357 | −3.926 | **< .001** |

**Table S2.** Hierarchical regression analysis of predictors of psychopathology (measured by PANSS total score) in patients with schizophrenia, using number of childhood trauma types.

|  | *R* | *R^2^* | *∆R^2^* | *p* | *B* | *SE B* | β | *t* | *p* |
| --- | --- | --- | --- | --- | --- | --- | --- | --- | --- |
| **Model 1** | .116 | .013 |  | .344 |  |  |  |  |  |
| Sex |  |  |  |  | −2.795 | 2.806 | −0.080 | −0.996 | .321 |
| Age |  |  |  |  | −0.177 | 0.147 | −0.096 | −1.205 | .230 |
| **Model 2** | .715 | .512 | .498 | **< .001** |  |  |  |  |  |
| Sex |  |  |  |  | −1.512 | 1.983 | −0.043 | −0.762 | .447 |
| Age |  |  |  |  | 0.024 | 0.105 | 0.013 | 0.227 | .821 |
| Number of childhood trauma types |  |  |  |  | 6.475 | 0.513 | 0.714 | 12.613 | **< .001** |
| **Model 3** | .718 | .516 | .005 | .232 |  |  |  |  |  |
| Sex |  |  |  |  | −1.477 | 1.981 | −0.042 | −0.746 | .457 |
| Age |  |  |  |  | 0.001 | 0.106 | 0.001 | 0.010 | .992 |
| Number of childhood trauma types |  |  |  |  | 6.401 | 0.516 | 0.706 | 12.399 | **< .001** |
| Education years |  |  |  |  | −0.394 | 0.328 | −.069 | −1.201 | .232 |
| **Model 4** | .725 | .526 | .010 | .080 |  |  |  |  |  |
| Sex |  |  |  |  | −2.066 | 1.996 | −0.059 | −1.035 | .302 |
| Age |  |  |  |  | 0.043 | 0.108 | 0.024 | 0.398 | .691 |
| Number of childhood trauma types |  |  |  |  | 6.298 | 0.516 | 0.695 | 12.201 | **< .001** |
| Education years |  |  |  |  | −0.471 | 0.329 | −0.082 | −1.432 | .154 |
| Age of schizophrenia onset |  |  |  |  | −0.217 | 0.124 | −0.105 | −1.760 | .080 |
| **Model 5** | .731 | .534 | .008 | .257 |  |  |  |  |  |
| Sex |  |  |  |  | −2.046 | 2.000 | −0.058 | −1.023 | .308 |
| Age |  |  |  |  | 0.048 | 0.108 | 0.026 | 0.445 | .657 |
| Number of childhood trauma types |  |  |  |  | 6.232 | 0.518 | 0.688 | 12.037 | **< .001** |
| Education years |  |  |  |  | −0.507 | 0.331 | −0.088 | −1.530 | .128 |
| Age of schizophrenia onset |  |  |  |  | −0.244 | 0.126 | −0.117 | −1.932 | .055 |
| Antipsychotic dose |  |  |  |  | 0.005 | 0.003 | 0.089 | 1.573 | .118 |
| MMSE score |  |  |  |  | −0.564 | 1.011 | −0.032 | −0.558 | .578 |
| **Model 6** | .776 | .603 | .069 | **< .001** |  |  |  |  |  |
| Sex |  |  |  |  | −1.907 | 1.853 | −.054 | −1.029 | .305 |
| Age |  |  |  |  | .017 | .100 | .009 | .169 | .866 |
| Number of childhood trauma types |  |  |  |  | 4.084 | .638 | .451 | 6.402 | **< .001** |
| Education years |  |  |  |  | −.229 | .312 | −.040 | −.735 | .464 |
| Age of schizophrenia onset |  |  |  |  | −.183 | .117 | −.088 | −1.558 | .121 |
| Antipsychotic dose |  |  |  |  | .005 | .003 | .086 | 1.636 | .104 |
| MMSE score |  |  |  |  | .307 | .952 | .017 | .322 | .748 |
| Plasma oxytocin level |  |  |  |  | −64.727 | 12.667 | −.369 | −5.110 | **< .001** |

**Table S3.** Mediation effect of plasma oxytocin level on the relationship between childhood trauma and psychopathology (measured by PANSS total score) in patients with schizophrenia, using number of childhood trauma types.

Model Summary

|  | *R* | *R^2^* | *MSE* | *F* | *p* |
| --- | --- | --- | --- | --- | --- |
| Model 4 | .776 | .603 | 124.080 | 28.634 | < .001 |

Covariates: sex, age, year of education, MMSE score, age of schizophrenia onset, antipsychotic dose.

Mediation Estimates

| Effect | Estimate | *SE* | 95% CI | % Mediation |
| --- | --- | --- | --- | --- |
| Indirect | 2.149 | 0.450 | [1.344, 3.094] | 34.48 |
| Direct | 4.084 | 0.638 | [2.823, 5.344] | 65.52 |
| Total | 6.233 | 0.518 | [5.210, 7.255] | 100.00 |

Path Estimates

|  |  |  | Estimate | *SE* | *t* | *p*^†^ | |
| --- | --- | --- | --- | --- | --- | --- | --- |
| Number of childhood trauma types | 🡪 | Oxytocin level | −0.033 | 0.003 | −10.808 | < .001 | |
| Oxytocin level | 🡪 | PANSS total score | −64.727 | 12.667 | −5.110 | < .001 | |
| Number of childhood trauma types | 🡪 | PANSS total score | 4.084 | 0.638 | 6.402 | < .001 | |
| ^†^ Bonferroni-corrected *p* value | | | | | | |  |

**Table S4.** Summary of theoretical exploratory analyses for mediation models.

| Model | X | M | Y | X🡪M | M🡪Y | X🡪Y | R^2^ | Mod |
| --- | --- | --- | --- | --- | --- | --- | --- | --- |
| 0 | CTQ | Oxytocin | PANSS | < .001 | < .001 | < .001 | < .001 | Y |
| 1 | Physical abuse | Oxytocin | PANSS Total | < .001 | < .001 | < .001 | < .001 | Y |
| 2 | Physical abuse | Oxytocin | PANSS Positive | < .001 | .924 | .030 | .020 | N |
| 3 | Physical abuse | Oxytocin | PANSS Negative | < .001 | < .001 | .021 | < .001 | Y |
| 4 | Physical abuse | Oxytocin | PANSS General | < .001 | < .001 | .006 | < .001 | Y |
| 5 | Emotional abuse | Oxytocin | PANSS Total | < .001 | < .001 | < .001 | < .001 | Y |
| 6 | Emotional abuse | Oxytocin | PANSS Positive | < .001 | .995 | .060 | .032 | N |
| 7 | Emotional abuse | Oxytocin | PANSS Negative | < .001 | < .001 | .018 | < .001 | Y |
| 8 | Emotional abuse | Oxytocin | PANSS General | < .001 | < .001 | < .001 | < .001 | Y |
| 9 | Sexual abuse | Oxytocin | PANSS Total | < .001 | < .001 | .092 | .001 | Y |
| 10 | Sexual abuse | Oxytocin | PANSS Positive | < .001 | .039 | .387 | .831 | Y |
| 11 | Sexual abuse | Oxytocin | PANSS Negative | < .001 | < .001 | .193 | < .001 | Y |
| 12 | Sexual abuse | Oxytocin | PANSS General | < .001 | < .001 | .575 | .001 | Y |
| 13 | Physical neglect | Oxytocin | PANSS Total | < .001 | < .001 | .002 | < .001 | Y |
| 14 | Physical neglect | Oxytocin | PANSS Positive | < .001 | .415 | .144 | .136 | N |
| 15 | Physical neglect | Oxytocin | PANSS Negative | < .001 | < .001 | .023 | < .001 | Y |
| 16 | Physical neglect | Oxytocin | PANSS General | < .001 | < .001 | .013 | < .001 | Y |
| 17 | Emotional neglect | Oxytocin | PANSS Total | < .001 | < .001 | < .001 | < .001 | Y |
| 18 | Emotional neglect | Oxytocin | PANSS Positive | < .001 | .837 | .008 | .008 | N |
| 19 | Emotional neglect | Oxytocin | PANSS Negative | < .001 | < .001 | .005 | < .001 | Y |
| 20 | Emotional neglect | Oxytocin | PANSS General | < .001 | < .001 | < .001 | < .001 | Y |

Model 0 Summary

|  | *R* | *R^2^* | *MSE* | *F* | *p* |
| --- | --- | --- | --- | --- | --- |
| Model 4 | .719 | .517 | 150.014 | 23.195 | < .001 |

Covariates: sex, age, year of education, MMSE, age of onset, antipsychotic dose.

Mediation Estimates

| Effect | Estimate | *SE* | 95% CI | % Mediation |
| --- | --- | --- | --- | --- |
| Indirect | 0.183 | 0.044 | [0.102, 0.272] | 38.36 |
| Direct | 0.294 | 0.058 | [0.180, 0.409] | 61.64 |
| Total | 0.477 | 0.041 | [0.400, 0.559] | 100.00 |

Path Estimates

|  |  |  | Estimate | *SE* | *t* | *p* |
| --- | --- | --- | --- | --- | --- | --- |
| CTQ | 🡪 | Oxytocin | −0.003 | 0.001 | −13.522 | < .001 |
| Oxytocin | 🡪 | PANSS | −62.896 | 14.733 | −4.269 | < .001 |
| CTQ | 🡪 | PANSS | 0.295 | 0.058 | 5.079 | < .001 |

Model 1 Summary

|  | *R* | *R^2^* | *MSE* | *F* | *p* |
| --- | --- | --- | --- | --- | --- |
| Model 4 | .676 | .457 | 167.395 | 21.454 | < .001 |

Covariates: sex, age, year of education, MMSE, age of onset, antipsychotic dose.

Mediation Estimates

| Effect | Estimate | *SE* | 95% CI | % Mediation |
| --- | --- | --- | --- | --- |
| Indirect | 1.002 | 0.186 | [0.656, 1.390] | 46.09 |
| Direct | 1.172 | 0.269 | [0.641, 1.702] | 53.91 |
| Total | 2.174 | 0.210 | [1.760, 2.588] | 100.00 |

Path Estimates

|  |  |  | Estimate | *SE* | *t* | *p* |
| --- | --- | --- | --- | --- | --- | --- |
| PA | 🡪 | Oxytocin | −0.013 | 0.001 | −11.982 | < .001 |
| Oxytocin | 🡪 | PANSS | −75.926 | 14.158 | −5.363 | < .001 |
| PA | 🡪 | PANSS | 1.172 | 0.269 | 4.364 | < .001 |

Model 2 Summary

|  | *R* | *R^2^* | *MSE* | *F* | *p* |
| --- | --- | --- | --- | --- | --- |
| Model 4 | .304 | .092 | 45.914 | 2.595 | .020 |

Covariates: sex, age, year of education, MMSE, age of onset, antipsychotic dose.

Mediation Estimates

| Effect | Estimate | *SE* | 95% CI | % Mediation |
| --- | --- | --- | --- | --- |
| Indirect | −0.010 | 0.110 | [−0.228, 0.202] | −2.56 |
| Direct | 0.400 | 0.153 | [0.097, 0.703] | 102.56 |
| Total | 0.390 | 0.110 | [0.173, 0.607] | 100.00 |

Path Estimates

|  |  |  | Estimate | *SE* | *t* | *p* |
| --- | --- | --- | --- | --- | --- | --- |
| PA | 🡪 | Oxytocin | −0.013 | 0.001 | −11.982 | < .001 |
| Oxytocin | 🡪 | Pos | 0.774 | 8.086 | 0.096 | .924 |
| PA | 🡪 | Pos | 0.400 | 0.153 | 2.608 | .030 |

Model 3 Summary

|  | *R* | *R^2^* | *MSE* | *F* | *p* |
| --- | --- | --- | --- | --- | --- |
| Model 4 | .609 | .371 | 42.352 | 15.039 | < .001 |

Covariates: sex, age, year of education, MMSE, age of onset, antipsychotic dose.

Mediation Estimates

| Effect | Estimate | *SE* | 95% CI | % Mediation |
| --- | --- | --- | --- | --- |
| Indirect | 0.483 | 0.106 | [0.284, 0.710] | 56.62 |
| Direct | 0.370 | 0.136 | [0.101, 0.638] | 43.38 |
| Total | 0.853 | 0.105 | [0.644, 1.061] | 100.00 |

Path Estimates

|  |  |  | Estimate | *SE* | *t* | *p* |
| --- | --- | --- | --- | --- | --- | --- |
| PA | 🡪 | Oxytocin | −0.013 | 0.001 | −11.982 | < .001 |
| Oxytocin | 🡪 | Neg | −36.631 | 7.175 | −5.105 | < .001 |
| PA | 🡪 | Neg | 0.370 | 0.136 | 2.715 | .021 |

Model 4 Summary

|  | *R* | *R^2^* | *MSE* | *F* | *p* |
| --- | --- | --- | --- | --- | --- |
| Model 4 | .619 | .383 | 36.416 | 15.860 | < .001 |

Covariates: sex, age, year of education, MMSE, age of onset, antipsychotic dose.

Mediation Estimates

| Effect | Estimate | *SE* | 95% CI | % Mediation |
| --- | --- | --- | --- | --- |
| Indirect | 0.443 | 0.089 | [0.271, 0.624] | 53.12 |
| Direct | 0.391 | 0.126 | [0.141, 0.640] | 46.88 |
| Total | 0.834 | 0.098 | [0.641, 1.027] | 100.00 |

Path Estimates

|  |  |  | Estimate | *SE* | *t* | *p* |
| --- | --- | --- | --- | --- | --- | --- |
| PA | 🡪 | Oxytocin | −0.013 | 0.001 | −11.982 | < .001 |
| Oxytocin | 🡪 | Gen | −33.587 | 6.666 | −5.038 | < .001 |
| PA | 🡪 | Gen | 0.390 | 0.126 | 3.088 | .006 |

Model 5 Summary

|  | *R* | *R^2^* | *MSE* | *F* | *p* |
| --- | --- | --- | --- | --- | --- |
| Model 4 | .684 | .468 | 163.985 | 22.431 | < .001 |

Covariates: sex, age, year of education, MMSE, age of onset, antipsychotic dose.

Mediation Estimates

| Effect | Estimate | *SE* | 95% CI | % Mediation |
| --- | --- | --- | --- | --- |
| Indirect | 0.686 | 0.134 | [0.435, 0.954] | 44.95 |
| Direct | 0.840 | 0.190 | [0.464, 1.216] | 55.05 |
| Total | 1.526 | 0.144 | [1.242, 1.810] | 100.00 |

Path Estimates

|  |  |  | Estimate | *SE* | *t* | *p* |
| --- | --- | --- | --- | --- | --- | --- |
| EA | 🡪 | Oxytocin | −0.009 | 0.001 | −12.596 | < .001 |
| Oxytocin | 🡪 | PANSS | −73.253 | 14.495 | −5.054 | < .001 |
| EA | 🡪 | PANSS | 0.840 | 0.190 | 4.415 | < .001 |

Model 6 Summary

|  | *R* | *R^2^* | *MSE* | *F* | *p* |
| --- | --- | --- | --- | --- | --- |
| Model 4 | .292 | .085 | 46.285 | 2.371 | .032 |

Covariates: sex, age, year of education, MMSE, age of onset, antipsychotic dose.

Mediation Estimates

| Effect | Estimate | *SE* | 95% CI | % Mediation |
| --- | --- | --- | --- | --- |
| Indirect | −0.001 | 0.078 | [−0.150, 0.155] | −0.39 |
| Direct | 0.257 | 0.109 | [0.041, 0.472] | 100.39 |
| Total | 0.256 | 0.076 | [0.105, 0.407] | 100.00 |

Path Estimates

|  |  |  | Estimate | *SE* | *t* | *p* |
| --- | --- | --- | --- | --- | --- | --- |
| EA | 🡪 | Oxytocin | −0.009 | 0.001 | −12.596 | < .001 |
| Oxytocin | 🡪 | Pos | 0.056 | 8.323 | 0.007 | .995 |
| EA | 🡪 | Pos | 0.257 | 0.109 | 2.350 | .060 |

Model 7 Summary

|  | *R* | *R^2^* | *MSE* | *F* | *p* |
| --- | --- | --- | --- | --- | --- |
| Model 4 | .586 | .344 | 44.179 | 13.363 | < .001 |

Covariates: sex, age, year of education, MMSE, age of onset, antipsychotic dose.

Mediation Estimates

| Effect | Estimate | *SE* | 95% CI | % Mediation |
| --- | --- | --- | --- | --- |
| Indirect | 0.377 | 0.074 | [0.233, 0.521] | 67.32 |
| Direct | 0.183 | 0.098 | [−0.010, 0.376] | 32.68 |
| Total | 0.560 | 0.075 | [0.412, 0.707] | 100.00 |

Path Estimates

|  |  |  | Estimate | *SE* | *t* | *p* |
| --- | --- | --- | --- | --- | --- | --- |
| EA | 🡪 | Oxytocin | −0.009 | 0.001 | −12.596 | < .001 |
| Oxytocin | 🡪 | Neg | −40.255 | 7.447 | −5.406 | < .001 |
| EA | 🡪 | Neg | 0.183 | 0.100 | 1.869 | .018 |

Model 8 Summary

|  | *R* | *R^2^* | *MSE* | *F* | *p* |
| --- | --- | --- | --- | --- | --- |
| Model 4 | .653 | .427 | 33.864 | 18.977 | < .001 |

Covariates: sex, age, year of education, MMSE, age of onset, antipsychotic dose.

Mediation Estimates

| Effect | Estimate | *SE* | 95% CI | % Mediation |
| --- | --- | --- | --- | --- |
| Indirect | 0.273 | 0.066 | [0.142, 0.404] | 44.17 |
| Direct | 0.345 | 0.088 | [0.171, 0.519] | 55.83 |
| Total | 0.618 | 0.065 | [0.489, 0.747] | 100.00 |

Path Estimates

|  |  |  | Estimate | *SE* | *t* | *p* |
| --- | --- | --- | --- | --- | --- | --- |
| EA | 🡪 | Oxytocin | −0.009 | 0.001 | −12.596 | < .001 |
| Oxytocin | 🡪 | Gen | −29.160 | 6.715 | −4.343 | < .001 |
| EA | 🡪 | Gen | 0.345 | 0.088 | 3.914 | < .001 |

Model 9 Summary

|  | *R* | *R^2^* | *MSE* | *F* | *p* |
| --- | --- | --- | --- | --- | --- |
| Model 4 | .389 | .152 | 261.477 | 4.560 | .001 |

Covariates: sex, age, year of education, MMSE, age of onset, antipsychotic dose.

Mediation Estimates

| Effect | Estimate | *SE* | 95% CI | % Mediation |
| --- | --- | --- | --- | --- |
| Indirect | 1.536 | 0.223 | [1.179, 2.049] | 102.88 |
| Direct | −0.043 | 0.350 | [−0.734, 0.648] | −2.88 |
| Total | 1.493 | 0.402 | [0.698, 2.287] | 100.00 |

Path Estimates

|  |  |  | Estimate | *SE* | *t* | *p* |
| --- | --- | --- | --- | --- | --- | --- |
| SA | 🡪 | Oxytocin | −0.013 | 0.002 | −6.083 | < .001 |
| Oxytocin | 🡪 | PANSS | −119.571 | 12.021 | −9.947 | < .001 |
| SA | 🡪 | PANSS | −0.043 | 0.350 | −0.123 | .902 |

Model 10 Summary

|  | *R* | *R^2^* | *MSE* | *F* | *p* |
| --- | --- | --- | --- | --- | --- |
| Model 4 | .134 | .018 | 49.674 | 0.469 | .831 |

Covariates: sex, age, year of education, MMSE, age of onset, antipsychotic dose.

Mediation Estimates

| Effect | Estimate | *SE* | 95% CI | % Mediation |
| --- | --- | --- | --- | --- |
| Indirect | 0.211 | 0.094 | [0.062, 0.427] | 479.55 |
| Direct | −0.167 | 0.192 | [−0.546, 0.213] | −379.55 |
| Total | 0.044 | 0.175 | [−0.392, 0.391] | 100.00 |

Path Estimates

|  |  |  | Estimate | *SE* | *t* | *p* |
| --- | --- | --- | --- | --- | --- | --- |
| SA | 🡪 | Oxytocin | −0.013 | 0.002 | −6.083 | < .001 |
| Oxytocin | 🡪 | Pos | −16.424 | 6.599 | −2.489 | .039 |
| SA | 🡪 | Pos | −0.167 | 0.192 | −0.868 | .387 |

Model 11 Summary

|  | *R* | *R^2^* | *MSE* | *F* | *p* |
| --- | --- | --- | --- | --- | --- |
| Model 4 | .457 | .208 | 53.287 | 6.720 | < .001 |

Covariates: sex, age, year of education, MMSE, age of onset, antipsychotic dose.

Mediation Estimates

| Effect | Estimate | *SE* | 95% CI | % Mediation |
| --- | --- | --- | --- | --- |
| Indirect | 0.602 | 0.094 | [0.445, 0.824] | 73.06 |
| Direct | 0.222 | 0.170 | [−0.114, 0.559] | 26.94 |
| Total | 0.824 | 0.182 | [0.465, 1.182] | 100.00 |

Path Estimates

|  |  |  | Estimate | *SE* | *t* | *p* |
| --- | --- | --- | --- | --- | --- | --- |
| SA | 🡪 | Oxytocin | −0.013 | 0.003 | −6.083 | < .001 |
| Oxytocin | 🡪 | Neg | −46.813 | 5.848 | −8.005 | < .001 |
| SA | 🡪 | Neg | 0.222 | 0.170 | 1.307 | .193 |

Model 12 Summary

|  | *R* | *R^2^* | *MSE* | *F* | *p* |
| --- | --- | --- | --- | --- | --- |
| Model 4 | .379 | .143 | 50.601 | 4.266 | .001 |

Covariates: sex, age, year of education, MMSE, age of onset, antipsychotic dose.

Mediation Estimates

| Effect | Estimate | *SE* | 95% CI | % Mediation |
| --- | --- | --- | --- | --- |
| Indirect | 0.633 | 0.099 | [0.484, 0.867] | 116.57 |
| Direct | −0.090 | 0.160 | [−0.406, 0.226] | −16.57 |
| Total | 0.543 | 0.177 | [0.193, 0.892] | 100.00 |

Path Estimates

|  |  |  | Estimate | *SE* | *t* | *p* |
| --- | --- | --- | --- | --- | --- | --- |
| SA | 🡪 | Oxytocin | −0.013 | 0.002 | −6.083 | < .001 |
| Oxytocin | 🡪 | Gen | −49.275 | 5.495 | −8.967 | < .001 |
| SA | 🡪 | Gen | −0.090 | 0.160 | −0.563 | .575 |

Model 13 Summary

|  | *R* | *R^2^* | *MSE* | *F* | *p* |
| --- | --- | --- | --- | --- | --- |
| Model 4 | .630 | .396 | 186.047 | 16.747 | < .001 |

Covariates: sex, age, year of education, MMSE, age of onset, antipsychotic dose.

Mediation Estimates

| Effect | Estimate | *SE* | 95% CI | % Mediation |
| --- | --- | --- | --- | --- |
| Indirect | 1.069 | 0.197 | [0.718, 1.485] | 54.68 |
| Direct | 0.886 | 0.256 | [0.381, 1.391] | 45.32 |
| Total | 1.955 | 0.217 | [1.527, 2.383] | 100.00 |

Path Estimates

|  |  |  | Estimate | *SE* | *t* | *p* |
| --- | --- | --- | --- | --- | --- | --- |
| PN | 🡪 | Oxytocin | −0.012 | 0.001 | −10.744 | < .001 |
| Oxytocin | 🡪 | PANSS | −87.674 | 13.757 | −6.373 | < .001 |
| PN | 🡪 | PANSS | 0.886 | 0.256 | 3.463 | .002 |

Model 14 Summary

|  | *R* | *R^2^* | *MSE* | *F* | *p* |
| --- | --- | --- | --- | --- | --- |
| Model 4 | .247 | .061 | 47.504 | 1.656 | .136 |

Covariates: sex, age, year of education, MMSE, age of onset, antipsychotic dose.

Mediation Estimates

| Effect | Estimate | *SE* | 95% CI | % Mediation |
| --- | --- | --- | --- | --- |
| Indirect | 0.078 | 0.103 | [−0.113, 0.288] | 26.80 |
| Direct | 0.213 | 0.145 | [−0.074, 0.500] | 73.20 |
| Total | 0.291 | 0.110 | [0.075, 0.507] | 100.00 |

Path Estimates

|  |  |  | Estimate | *SE* | *t* | *p* |
| --- | --- | --- | --- | --- | --- | --- |
| PN | 🡪 | Oxytocin | −0.012 | 0.001 | −10.744 | < .001 |
| Oxytocin | 🡪 | Pos | −6.381 | 7.808 | −0.817 | .415 |
| PN | 🡪 | Pos | 0.213 | 0.145 | 1.468 | .144 |

Model 15 Summary

|  | *R* | *R^2^* | *MSE* | *F* | *p* |
| --- | --- | --- | --- | --- | --- |
| Model 4 | .594 | .353 | 43.593 | 13.886 | < .001 |

Covariates: sex, age, year of education, MMSE, age of onset, antipsychotic dose.

Mediation Estimates

| Effect | Estimate | *SE* | 95% CI | % Mediation |
| --- | --- | --- | --- | --- |
| Indirect | 0.465 | 0.103 | [0.272, 0.676] | 57.62 |
| Direct | 0.342 | 0.127 | [0.091, 0.593] | 42.38 |
| Total | 0.807 | 0.105 | [0.600, 1.014] | 100.00 |

Path Estimates

|  |  |  | Estimate | *SE* | *t* | *p* |
| --- | --- | --- | --- | --- | --- | --- |
| PN | 🡪 | Oxytocin | −0.012 | 0.001 | −10.744 | < .001 |
| Oxytocin | 🡪 | Neg | −38.116 | 6.829 | −5.582 | < .001 |
| PN | 🡪 | Neg | 0.342 | 0.127 | 2.695 | .023 |

Model 16 Summary

|  | *R* | *R^2^* | *MSE* | *F* | *p* |
| --- | --- | --- | --- | --- | --- |
| Model 4 | .597 | .357 | 38.006 | 14.130 | < .001 |

Covariates: sex, age, year of education, MMSE, age of onset, antipsychotic dose.

Mediation Estimates

| Effect | Estimate | *SE* | 95% CI | % Mediation |
| --- | --- | --- | --- | --- |
| Indirect | 0.437 | 0.087 | [0.280, 0.622] | 56.03 |
| Direct | 0.343 | 0.118 | [0.108, 0.575] | 43.97 |
| Total | 0.780 | 0.098 | [0.585, 0.972] | 100.00 |

Path Estimates

|  |  |  | Estimate | *SE* | *t* | *p* |
| --- | --- | --- | --- | --- | --- | --- |
| PN | 🡪 | Oxytocin | −0.012 | 0.001 | −10.744 | < .001 |
| Oxytocin | 🡪 | Gen | −35.866 | 6.366 | −5.634 | < .001 |
| PN | 🡪 | Gen | 0.342 | 0.118 | 2.885 | .013 |

Model 17 Summary

|  | *R* | *R^2^* | *MSE* | *F* | *p* |
| --- | --- | --- | --- | --- | --- |
| Model 4 | .714 | 0.511 | 150.766 | 26.633 | < .001 |

Covariates: sex, age, year of education, MMSE, age of onset, antipsychotic dose.

Mediation Estimates

| Effect | Estimate | *SE* | 95% CI | % Mediation |
| --- | --- | --- | --- | --- |
| Indirect | 0.627 | 0.124 | [0.392, 0.875] | 36.43 |
| Direct | 1.094 | 0.182 | [0.735, 1.453] | 63.57 |
| Total | 1.721 | 0.147 | [1.430, 2.013] | 100.00 |

Path Estimates

|  |  |  | Estimate | *SE* | *t* | *p* |
| --- | --- | --- | --- | --- | --- | --- |
| EN | 🡪 | Oxytocin | −0.009 | 0.001 | −10.924 | < .001 |
| Oxytocin | 🡪 | PANSS | −67.390 | 12.934 | 05.210 | < .001 |
| EN | 🡪 | PANSS | 1.094 | 0.182 | 6.018 | < .001 |

Model 18 Summary

|  | *R* | *R^2^* | *MSE* | *F* | *p* |
| --- | --- | --- | --- | --- | --- |
| Model 4 | .326 | .106 | 45.226 | 3.023 | .008 |

Covariates: sex, age, year of education, MMSE, age of onset, antipsychotic dose.

Mediation Estimates

| Effect | Estimate | *SE* | 95% CI | % Mediation |
| --- | --- | --- | --- | --- |
| Indirect | −0.015 | 0.074 | [−0.158, 0.131] | −4.78 |
| Direct | 0.329 | 0.1081 | [0.115, 0.542] | 104.78 |
| Total | 0.314 | 0.081 | [0.155, 0.474] | 100.00 |

Path Estimates

|  |  |  | Estimate | *SE* | *t* | *p* |
| --- | --- | --- | --- | --- | --- | --- |
| EN | 🡪 | Oxytocin | −0.009 | 0.001 | −10.924 | < .001 |
| Oxytocin | 🡪 | Pos | 1.588 | 7.689 | 0.206 | .837 |
| EN | 🡪 | Pos | 0.329 | 0.108 | 3.042 | .008 |

Model 19 Summary

|  | *R* | *R^2^* | *MSE* | *F* | *p* |
| --- | --- | --- | --- | --- | --- |
| Model 4 | .614 | .377 | 41.947 | 15.431 | < .001 |

Covariates: sex, age, year of education, MMSE, age of onset, antipsychotic dose.

Mediation Estimates

| Effect | Estimate | *SE* | 95% CI | % Mediation |
| --- | --- | --- | --- | --- |
| Indirect | 0.333 | 0.074 | [0.190, 0.480] | 52.11 |
| Direct | 0.306 | 0.096 | [0.117, 0.496] | 47.89 |
| Total | 0.639 | 0.078 | [0.486, 0.793] | 100.00 |

Path Estimates

|  |  |  | Estimate | *SE* | *t* | *p* |
| --- | --- | --- | --- | --- | --- | --- |
| EN | 🡪 | Oxytocin | −0.009 | 0.001 | −10.924 | < .001 |
| Oxytocin | 🡪 | Neg | −35.754 | 6.815 | −5.245 | < .001 |
| EN | 🡪 | Neg | 0.306 | 0.096 | 3.199 | .005 |

Model 20 Summary

|  | *R* | *R^2^* | *MSE* | *F* | *p* |
| --- | --- | --- | --- | --- | --- |
| Model 4 | .661 | .437 | 33.248 | 19.802 | < .001 |

Covariates: sex, age, year of education, MMSE, age of onset, antipsychotic dose.

Mediation Estimates

| Effect | Estimate | *SE* | 95% CI | % Mediation |
| --- | --- | --- | --- | --- |
| Indirect | 0.269 | 0.061 | [0.158, 0.395] | 40.03 |
| Direct | 0.403 | 0.087 | [0.232, 0.574] | 59.97 |
| Total | 0.672 | 0.069 | [0.535, 0.809] | 100.00 |

Path Estimates

|  |  |  | Estimate | *SE* | *t* | *p* |
| --- | --- | --- | --- | --- | --- | --- |
| EN | 🡪 | Oxytocin | −0.009 | 0.001 | −10.924 | < .001 |
| Oxytocin | 🡪 | Gen | −28.946 | 6.162 | −4.698 | < .001 |
| EN | 🡪 | Gen | 0.403 | 0.087 | 4.650 | < .001 |
